# The association of body-mass index and depressed mood with knee pain and activity limitations in knee osteoarthritis: results from the Amsterdam osteoarthritis cohort

**DOI:** 10.1186/1471-2474-14-296

**Published:** 2013-10-17

**Authors:** Jasmijn FM Holla, Marike van der Leeden, Dirk L Knol, Leo D Roorda, Martin van der Esch, Ramon E Voorneman, Willem F Lems, Joost Dekker

**Affiliations:** 1 Amsterdam Rehabilitation Research Centre | Reade, PO Box 58271, 1040 HG Amsterdam, The Netherlands; 2 Department of Rehabilitation Medicine, VU University Medical Centre, Amsterdam, The Netherlands; 3 EMGO Institute for Health and Care Research, VU University Medical Centre, Amsterdam, The Netherlands; 4 Department of Epidemiology and Biostatistics, VU University Medical Centre, Amsterdam, The Netherlands; 5 Jan van Breemen Research Institute | Reade, Amsterdam, The Netherlands; 6 Department of Rheumatology, VU University Medical Centre, Amsterdam, The Netherlands; 7 Department of Psychiatry, VU University Medical Centre, Amsterdam, The Netherlands

**Keywords:** Activity limitations, Body-mass index, Depressed mood, Knee, Osteoarthritis, Pain

## Abstract

**Background:**

Body-mass index (BMI) and depressed mood are both positively associated with pain and activity limitations in knee osteoarthritis (OA), and are interrelated. The aims of the present study were: 1) to assess whether BMI and depressed mood are independently associated with knee pain and activity limitations; and 2) to compare the relative contributions of BMI and depressed mood to knee pain and activity limitations.

**Methods:**

A cross-sectional study in 294 patients with clinical knee OA. Regression analyses were performed with knee pain or activity limitations (self-reported and performance-based) as dependent variables, and BMI and depressed mood as independent variables. All analyses were adjusted for age, gender, marital status, education level, radiographic OA and comorbidity. Dominance analyses were performed to examine the relative contributions of BMI and depressed mood to knee pain and activity limitations.

**Results:**

BMI and depressed mood were positively and independently associated with knee pain and activity limitations. BMI and depressed mood explained small parts (3.0% and 2.3%, respectively) of variance in knee pain. BMI explained a substantial part of variance in both self-reported (9.8%) and performance-based (20.4%) activity limitations, while depressed mood explained a small part of variance (3.1% in self-reported and 2.6% in performance-based activity limitations).

**Conclusions:**

In patients with knee OA both BMI and depressed mood seem to be independently associated with knee pain and activity limitations. The contribution of BMI to activity limitations is most substantial, thereby offering a relevant target for interventions.

## Background

Overweight and depressed mood are more common in knee osteoarthritis (OA) than in the general population [1-3], and are both positively associated with pain and activity limitations [4-13]. There is growing evidence that overweight, as indicated by body-mass index (BMI) ≥ 25 kg/m^2^, and depressed mood are interrelated [14,15], and that common biological and psychological mechanisms underlie the development of both overweight and depressed mood [16]. Therefore, the question arises whether BMI and depressed mood are independently associated with pain and activity limitations in knee OA. This information may guide the design of interventions targeting bodyweight or depressed mood as a means to improve pain and activity limitations in knee OA.

Two studies that examined a broad set of determinants of pain and activity limitations in patients with knee OA found that BMI and depressed mood were independently associated with pain and activity limitations [12,17]. On the other hand, three other studies did not find independent associations between BMI, depressed mood, pain and activity limitations [9,18,19]. The five studies described above were not primarily aimed at examining the interrelations between BMI, depressed mood, pain and activity limitations. Furthermore, they did not examine the relative contributions of BMI and depressed mood to pain and activity limitations. Thus, there is a need to examine these interrelations more thoroughly.

The aims of the present study were: 1) to assess whether BMI and depressed mood are independently associated with knee pain and activity limitations in patients with knee OA; and 2) to compare the relative contributions of BMI and depressed mood to knee pain and activity limitations.

## Methods

### Patients

A cross-sectional study was conducted in a sample of 294 patients from the Amsterdam Osteoarthritis (AMS-OA) cohort. Since 2009 all patients of 18 years or older with OA of the knee or hip who have been referred to Reade, an outpatient rheumatology rehabilitation centre in the Netherlands, are included in the AMS-OA cohort. Inclusion criteria were: clinical OA according to the American College of Rheumatology (ACR) criteria [20]. Exclusion criteria were: total knee replacement, and any other form of arthritis (e.g. crystal arthropathy, septic arthritis, spondylarthropathy). After inclusion, patients were assessed by rheumatologists, radiologists and rehabilitation physicians. Measurements included demographic, clinical, radiographic, neuromuscular and psychological factors. For the present study baseline data of patients recruited between January 2009 and January 2012 were used. The study was approved by the Medical Ethical Committee of Reade. All participants gave their written informed consent before entering the study.

### Measures

#### Knee pain

Knee pain was assessed with a numeric rating scale (NRS) for knee pain. The NRS consisted of a horizontal set of numbers, ranging from 0 to 10, anchored by word descriptors at each end ('no pain’ and 'worst imaginable pain’). The patient was asked to mark the number that indicated the intensity of the knee pain experienced during the last week. The NRS has been shown to be a reliable and valid measure of pain [21].

### Activity limitations

Activity limitations were assessed with both a self-report measure and a performance-based measure. Self-reported activity limitations were assessed with the physical function subscale of the Western Ontario and McMaster Universities Osteoarthritis Index (WOMAC-PF) [22,23]. This subscale consists of 17 items, which assess the degree of difficulties one has in executing activities. Items are answered on a 5-point scale. The sum of scores on all 17 items is used as score for activity limitations. Scores range from 0 to 68, with higher scores indicating greater activity limitations. The WOMAC is widely used in clinical research and has been shown to be reliable, valid and responsive for use in patients with OA [22-24].

Performance-based activity limitations were assessed with a get up and go (GUG) test [25]. Patients began the test by sitting in a standard-height chair with armrests. On the command “go” they stood up without help of their arms and walked a distance of 15 meters. They were instructed to walk as quickly as they felt safe and comfortable. A stopwatch was used to measure the time it took the patient to get up from the chair and walk 15 meters. A longer time to complete the test indicates greater activity limitations. Excellent inter- and intrarater reliability have been reported for a comparable GUG test in patients with knee OA [26].

### Body-mass index (BMI)

The weight in kilograms (kg) and height in meters (m) were measured in standing position. The BMI was calculated using the standard formula: kg/m^2^.

### Depressed mood

Depressed mood was measured with the depression subscale of the Hospital Anxiety and Depression Scale (HADS) [27]. The HADS is a simple self-report questionnaire that was developed to screen for depression and anxiety in medical practice [28]. The depression subscale consists of 7 items, which assess depressed mood. Items are answered on a 4-point scale. The sum of scores on all 7 items is used as score for depressed mood. Scores range from 0 to 21, with higher scores indicating a more depressed mood. The HADS is widely used in clinical research, and has been shown to be reliable, valid and responsive for use as a screening tool in patients with OA [1].

### Additional measures

Additional data recorded were age, gender, marital status, education level, the duration of knee symptoms, the ACR clinical criteria for knee OA [20], the Kellgren and Lawrence grade (KL-grade) for radiographic knee OA [29], and comorbidity measured with the Cumulative Illness Rating Scale (CIRS) [30].

### Statistical analysis

Linear regression analyses were performed to assess the associations between BMI, depressed mood, and knee pain and activity limitations. Separate analyses were conducted for the dependent variables knee pain (NRS knee pain), self-reported activity limitations (WOMAC-PF) and performance-based activity limitations (GUG test). Prior to the analyses we checked whether the assumptions for linear regression (e.g. no strong multicollinearity between independent variables [Pearson’s correlation coefficient (r) < 0.80], homoscedasticity, linearity) were met [31]. First, a regression analysis was performed with BMI as independent variable and knee pain as dependent variable (analysis 1). Second, a regression analysis was performed with depressed mood as independent variable and knee pain as dependent variable (analysis 2). Third, a regression analysis was performed with both BMI and depressed mood as independent variables and knee pain as dependent variable (analysis 3). In all analyses we adjusted for age, gender, marital status (single/living alone vs. married/living together), education level (primary or secondary school vs. higher professional education or university), radiographic OA (highest KL-grade < 2 vs. highest KL-grade ≥ 2 [29]) and comorbidity (CIRS total score [30]). BMI and depressed mood were considered to be both independently associated with knee pain if BMI and depressed mood in analysis 3 were significantly associated with knee pain. The squared semi-partial correlation coefficient (r^2^) was calculated as measure of the amount of variance in knee pain that is accounted for by BMI or depressed mood adjusted for the effect of the other independent variables in the analysis [31]. Results were considered statistically significant at p < 0.05.

Dominance analysis was performed to examine the relative contributions of BMI and depressed mood to the explanation of variance in knee pain [32]. For this purpose the semi-partial r^2^s of BMI in analyses 1 and 3 and semi-partial r^2^s of depressed mood in analyses 2 and 3 were averaged and compared. If the averaged semi-partial r^2^ of BMI is greater than the averaged semi-partial r^2^ of depressed mood, BMI contributes more to the explanation of variance in knee pain than depressed mood. The same analyses were performed with self-reported and performance-based activity limitations as dependent variables.

## Results

### Study population

Characteristics of the study population are presented in Table 1. The study population consisted of 294 patients, 188 women and 106 men, with a mean age of 61.1 years. Around half (56.2%) of patients reported a knee symptom duration > 5 years, and 72.1% had radiographic knee OA (i.e. KL-grade ≥ 2). The mean score on the NRS for knee pain was 5, the mean score for self-reported activity limitations (WOMAC-PF) was 25.1, and the mean score for performance-based activity limitations (GUG test) was 11.2 seconds. The mean BMI was 29.2 kg/m^2^ and the median HADS depression score was 3.

**Table 1:** Characteristics of the study population (n = 294)

**Characteristic**	**Value**	**Missing (%)**
*Demographics*		
Age (years), mean ± SD	61.1 ± 7.4	
Female, n (%)	188 (63.9)	
Marital status, n (%)		15 (5.1)
Single/living alone	54 (19.4)	
Married/living together	225 (80.6)	
Education level, n (%)		10 (3.4)
Primary school	12 (4.2)	
Secondary school	173 (60.9)	
Higher professional education/university	99 (34.9)	
*Clinical factors*		
Duration of symptoms, n (%)		18 (6.1)
0–1 year	31 (11.2)	
1–5 years	90 (32.6)	
> 5 years	155 (56.2)	
KL-grade < 2, n (%)	80 (27.9)	7 (2.4)
KL-grade ≥ 2 , n (%)	207 (72.1)	
right knee	28 (9.8)	
left knee	35 (12.2)	
both knees	144 (50.2)	
Comorbidity, CIRS total score (range: 0–52), median (IQR)	2 (1–4)	2 (0.7)
*Independent variables*		
BMI, kg/m^2^, mean ± SD	29.2 ± 5.5	
HADS depression score (range: 0–21), median (IQR)	3 (1–6)	
*Dependent variables*		
NRS knee pain last week(range: 0-10), mean ± SD	5.0 ± 2.2	4 (1.4)
WOMAC physical function score (range: 0–68), mean ± SD	25.1 ± 13.1	4 (1.4)
Get up and go test (sec.), mean ± SD	11.2 ± 5.0	1 (0.3)

*SD*, Standard deviation. *IQR*, Interquartile range. *KL-grade*, Kellgren and Lawrence grade. *CIRS*, Cumulative Illness Rating Scale. *BMI*, Body-mass index. *HADS*, Hospital Anxiety and Depression Scale. *NRS*, Numeric rating scale. *WOMAC*, Western Ontario and McMaster Universities Osteoarthritis Index.

### Associations between BMI, depressed mood and knee pain

The correlation between BMI and depressed mood was 0.21 (p < 0.001). The results of the multivariable analyses are presented in Table 2. BMI was positively and independently associated with the NRS for knee pain. In analysis 1, in which we adjusted for all covariates, the standardized regression coefficient (β) of BMI was 0.19 (p = 0.002). In analysis 3, in which we additionally adjusted for depressed mood, the β of BMI was 0.16 (p = 0.009).

**Table 2:** Association of BMI and depressed mood with knee pain

**Analysis**	**Independent variables**	**B (95% CI)**	**β**	**p**	**Semi-partial r**^**2**^**× 100%**
1.	BMI	0.07 (0.03, 0.12)	0.19	0.002	3.5%
2.	HADS depression	0.12 (0.04, 0.20)	0.17	0.005	2.9%
3.	BMI	0.06 (0.02, 0.11)	0.16	0.009	2.4%
	HADS depression	0.09 (0.01, 0.18)	0.14	0.025	1.8%
	**Dominance analysis**				
	Overall average BMI	(0.035 + 0.024)/2 = 0.030			3.0%
	Overall average HADS depression	(0.029 + 0.018)/2 = 0.023			2.3%

Knee pain was measured with a numeric rating scale for knee pain during the last week. In all analyses we adjusted for age, gender, marital status, education level, radiographic OA and comorbidity. *B (95% CI)*, regression coefficient ( 95% confidence interval). *β*, Standardized regression coefficient. *Semi-partial r*^*2*^, Squared semi-partial correlation coefficient. *BMI*, Body-mass index. *HADS*, Hospital Anxiety and Depression Scale.

Depressed mood was positively and independently associated with the NRS for knee pain. In analysis 2, in which we adjusted for all covariates, the β of depressed mood was 0.17 (p = 0.005). In analysis 3, in which we additionally adjusted for BMI, the β of depressed mood was 0.14 (p = 0.025).

Dominance analysis revealed that BMI contributed more to the explanation of variance in knee pain than depressed mood. The averaged semi-partial r^2^ of BMI was 3.0% and the averaged semi-partial r^2^ of depressed mood was 2.3% (Table 2).

### Associations between BMI, depressed mood and self-reported activity limitations

BMI was positively and independently associated with self-reported activity limitations (Table 3). In analysis 1, in which we adjusted for all covariates, the β of BMI was 0.33 (p < 0.001). In analysis 3, in which we additionally adjusted for depressed mood, the β of BMI was 0.30 (p < 0.001).

**Table 3:** Association of BMI and depressed mood with self-reported activity limitations

**Analysis**	**Independent variables**	**B (95% CI)**	**β**	**p**	**Semi-partial r**^**2**^**× 100%**
1.	BMI	0.77 (0.21, 1.03)	0.33	<0.001	10.9%
2.	HADS depression	0.83 (0.37, 1.29)	0.21	<0.001	4.2%
3.	BMI	0.70 (0.44, 0.96)	0.30	<0.001	8.7%
	HADS depression	0.59 (0.14, 1.04)	0.15	0.011	2.0%
	**Dominance analysis**				
	Overall average BMI	(0.109 + 0.087)/2 = 0.098			9.8%
	Overall average HADS depression	(0.042 + 0.020)/2 = 0.031			3.1%

Self-reported activity limitations were measured with the physical function subscale of the Western Ontario and McMaster Universities Osteoarthritis Index. In all analyses we adjusted for age, gender, marital status, education level, radiographic OA and comorbidity. *B (95% CI)*, Regression coefficient ( 95% confidence interval). *β*, Standardized regression coefficient. Semi-partial r^2^ = squared semi-partial correlation coefficient. *BMI*, Body-mass index. *HADS*, Hospital Anxiety and Depression Scale.

Depressed mood was positively and independently associated with self-reported activity limitations. In analysis 2, in which we adjusted for all covariates, the β of depressed mood was 0.21 (p < 0.001). In analysis 3, in which we additionally adjusted for BMI, the β of depressed mood was 0.15 (p = 0.011).

Dominance analysis revealed that BMI contributed more to the explanation of variance in self-reported activity limitations than depressed mood. The averaged semi-partial r^2^ of BMI was 9.8% and the averaged semi-partial r^2^ of depressed mood was 3.1% (Table 3).

### Associations between BMI, depressed mood and performance-based activity limitations

BMI was positively and independently associated with performance-based activity limitations (Table 4). In analysis 1, in which we adjusted for all covariates, the β of BMI was 0.47 (p < 0.001). In analysis 3, in which we additionally adjusted for depressed mood, the β of BMI was 0.45 (p < 0.001).

**Table 4:** Association of BMI and depressed mood with performance-based activity limitations

**Analysis**	**Independent variables**	**B (95% CI)**	**β**	**p**	**Semi-partial r**^**2**^**× 100%**
1.	BMI	0.42 (0.33, 0.51)	0.47	<0.001	21.9%
2.	HADS depression	0.31 (0.14, 0.48)	0.21	0.001	4.0%
3.	BMI	0.40 (0.31, 0.49)	0.45	<0.001	19.0%
	HADS depression	0.17 (0.01, 0.32)	0.11	0.038	1.1%
	**Dominance analysis**				
	Overall average BMI	(0.219 + 0.190)/2 = 0.204			20.4%
	Overall average HADS depression	(0.040 + 0.011)/2 = 0.026			2.6%

Performance-based activity limitations were assessed with a get up and go test. In all analyses we adjusted for age, gender, marital status, education level, radiographic OA and comorbidity. *B (95% CI)*, Regression coefficient ( 95% confidence interval). *β*, Standardized regression coefficient. *Semi-partial r*^*2*^, Squared semi-partial correlation coefficient. *BMI*, Body-mass index. *HADS*, Hospital Anxiety and Depression Scale.

Depressed mood was positively and independently associated with performance-based activity limitations. In analysis 2, in which we adjusted for all covariates, the β of depressed mood was 0.21 (p = 0.001). In analysis 3, in which we additionally adjusted for BMI, the β of depressed mood was 0.11 (p = 0.038).

Dominance analysis revealed that BMI contributed more to the explanation of variance in performance-based activity limitations than depressed mood. The averaged semi-partial r^2^ of BMI was 20.4% and the averaged semi-partial r^2^ of depressed mood was 2.6% (Table 4).

## Discussion

BMI and depressed mood were found to be positively and independently associated with knee pain and activity limitations. BMI and depressed mood explained small parts of variance in knee pain. BMI explained a substantial part of variance in activity limitations, while depressed mood made a small contribution.

This is the first study that is primarily aimed at examining the interrelations between BMI, depressed mood, knee pain and activity limitations in knee OA. Five earlier studies examined these interrelations in the context of a larger study on determinants of pain and activity limitations [9,12,17-19]. The results of two of these five studies were in agreement with our results [12,17]. Three studies did not find independent associations between BMI, depressed mood, and pain and activity limitations [9,18,19]. However, the latter studies found that BMI and helplessness [18] or anxiety [9] (psychological concepts closely related to depressed mood) were independently associated with pain or activity limitations. Possibly, although it is important to distinguish depressed mood, helplessness and anxiety, these concepts are not completely separable in empirical models. Therefore, although the results of these two studies [9,18] differ from our results, they seem not to be in conflict with our findings.

In the present study, BMI and depressed mood were independently associated with knee pain and activity limitations in patients with knee OA. This indicates that in clinical practice BMI and depressed mood should both be monitored and targeted. However, BMI and depressed mood explained only a small part of variance in knee pain, which suggests that treatment of these conditions may result in only modest improvement in pain. On the other hand, BMI explained a substantial part of variance in activity limitations, while depressed mood made a small contribution. This suggests that bodyweight is a relevant treatment target, resulting in improvement in activity limitations. Treatment of depressed mood is expected to result in only modest improvement in activity limitations. These predictions regarding the effects of interventions seem to be supported by the literature. Studies in patients with knee OA and overweight or obesity have shown that weight reduction interventions lead to small to large effect sizes with greater improvements in activity limitations than in pain [33-35]. Little is known about the effect of depression interventions in patients with knee OA. One large study compared the effect of collaborative depression care with that of usual depression care in patients with OA and a major depression or dysthymia, and reported small to moderate effect sizes for pain and activity limitations [36]. The results of secondary analyses in the latter study suggested that depression interventions could be improved by targeting not only depression, but also pain using a combined medication and behavioural approach [2,36].

Several mechanisms have been proposed to explain the associations between BMI, depressed mood, knee pain and activity limitations in patients with knee OA. Increased mechanical stress [5,6] may explain the association between BMI and knee pain and activity limitations. Overweight increases the load on the knees during weight bearing activities, which may lead to pain and activity limitations. Fatigue [7,37] may explain the association between depressed mood and knee pain and activity limitations. Fatigue is associated with depressed mood and pain [7], and may lead to decreased motor activity resulting in activity limitations [37]. The interrelations between BMI, depressed mood and knee pain may be explained by low-grade inflammation and dysregulation of the hypothalamic-pituitary-adrenal axis (HPA axis). Overweight and depressed mood have been associated with activation of inflammatory pathways, including increases in C-reactive protein [15,37]. The HPA-axis may be involved in such pathways [37], and chronic low-level inflammation may lead to pain. The interrelations between BMI, depressed mood and activity limitations may be explained by low self-efficacy (i.e. low confidence in the ability to complete a task or activity) [38,39]. In both overweight and depressed people low self-efficacy may lead to avoidance of activities [40,41] and thereby activity limitations [42]. The validity of these proposed mechanisms has not been examined in patients with knee OA and could be a target for further research.

Some methodological issues need to be addressed. First, in our study population the prevalence of depressed mood was rather low: only 11.2% had a HADS depression score > 7 indicating probable depressed mood [28]. Despite this low prevalence we found significant associations between depressed mood, knee pain and activity limitations. However, the low prevalence of depressed mood could have influenced the strength of the associations found. Therefore, more studies are needed to externally validate our findings. Second, because the present study had a cross-sectional design no causal inferences can be made. It is hypothesized that high BMI and depressed mood lead to knee pain and activity limitations, however reverse causation (i.e. knee pain and activity limitations lead to a high BMI and depressed mood) is also possible and cannot be ruled out. The results of the present study may guide further research aimed at unravelling the causal pathways and mechanisms underlying the interrelations between BMI, depressed mood, knee pain and activity limitations. For this purpose longitudinal studies are needed. Third, our suggestions about the possible effects of interventions targeting depressed mood and especially BMI are hypothetical and cannot be confirmed based on the results of the present study.

## Conclusions

BMI and depressed mood seem to be independently associated with knee pain and activity limitations in patients with knee OA. The contribution of BMI to activity limitations seems to be most substantial, thereby offering a relevant target for interventions.

## Competing interests

The authors declare that they have no competing interest.

## Authors’ contributions

All authors have made substantial contributions to all of the following: (1) the conception and design of the study (JFMH, MvdL, JD), or acquisition of data (LDR, MvdE, REV, WFL), or analysis and interpretation of data (JFMH, MvdL, DLK, JD) (2) drafting the article or revising it critically for important intellectual content, (3) final approval of the version to be submitted. All authors read and approved the final manuscript.

## Pre-publication history

The pre-publication history for this paper can be accessed here:

http://www.biomedcentral.com/1471-2474/14/296/prepub
